# A phase I dose-escalation study of SYHA1813, a VEGFR and CSF1R inhibitor, in patients with recurrent High-Grade Gliomas or Advanced Solid Tumors

**DOI:** 10.1007/s10637-022-01325-4

**Published:** 2023-03-08

**Authors:** Zhuang Kang, Shenglan Li, Yi Lin, Yongsheng Li, Ying Mao, Jing Zhang, Ting Lei, Haidan Wang, Yangzhi Su, Yang Yang, Jingbo Qiu, Wenbin Li

**Affiliations:** 1 Department of Neuro-Oncology, Cancer Center, Beijing Tiantan Hospital, Capital Medical University, Beijing, China; 2 Department of Medical Oncology, Chongqing University Cancer Hospital, Chongqing University, Chongqing, China; 3 Department of Neurosurgery, Huashan Hospital, Fudan University, Shanghai, China; 4 Phase I Clinical Research Center, Huashan Hospital, Fudan University, Shanghai, China; 5 Department of Neurosurgery, Tongji Hospital, Tongji Medical College, Huazhong University of Science & Technology, Wuhan, China; 6 Department of Clinical Development, CSPC Pharmaceutical Group Limited, Shijiazhuang, China

**Keywords:** VEGFR, CSF1R, Solid tumor, High-grade glioma, Phase I, Dose escalation

## Abstract

SYHA1813 is a potent multikinase inhibitor that targets vascular endothelial growth factor receptors (VEGFRs)/colony-stimulating factor 1 receptor (CSF1R). This study aimed to evaluate the safety, pharmacokinetics (PK), and antitumor activity of escalating doses of SYHA1813 in patients with recurrent high-grade gliomas (HGGs) or advanced solid tumors. This study adopted a combination of accelerated titration and a 3 + 3 design for dose escalation, with a starting dose of 5 mg once daily. The dose escalation continued at successive dose levels until the maximum tolerated dose (MTD) was determined. A total of 14 patients were enrolled and treated, including 13 with WHO grade III or IV gliomas and 1 with colorectal cancer. Two patients experienced dose-limiting toxicities (grade 4 hypertension and grade 3 mucositis oral) at 30 mg SYHA1813. The MTD was defined as 15 mg once daily. Hypertension (n = 6, 42.9%) was the most frequent treatment-related adverse event. Among evaluable patients (n = 10), 2 (20%) patients achieved partial response, and 7 (70%) had stable disease. The exposure increased with increasing doses within the studied dose range of 5 to 30 mg. Biomarker assessments demonstrated significant reductions in the levels of soluble VEGFR2 (*P* = .0023) and increases in the levels of VEGFA (*P* = .0092) and placental growth factor (*P* = .0484). The toxicities of SYHA1813 were manageable, and encouraging antitumor efficacy was observed in patients with recurrent malignant glioma. This study is registered with the Chinese Clinical Trial Registry (www.chictr.org.cn/index.aspx; identifier ChiCTR2100045380).

**Supplementary Information:**

The online version contains supplementary material available at 10.1007/s10637-022-01325-4.

## Introduction

Despite advances in treating recurrent high-grade glioma (HGG, WHO grade III and IV gliomas), the prognosis remains poor, and much work still needs to be done for improvement. Malignant tumors require neovascularization for growth, invasion, and metastasis[1]. Vascular endothelial growth factor (VEGF) and its receptors (VEGFR-1 to -3) play major roles in tumor angiogenesis[2]. As VEGFR is attractive target, various VEGFR tyrosine kinase inhibitors (TKIs) have been successfully approved for multiple types of cancer. However, none of these TKIs have been approved for patients with HGG, who may rapidly develop resistance to anti-VEGFR therapy with activation of compensatory angiogenesis, growth, and survival pathways[3, 4].

Tumor-associated macrophages (TAMs), defined as macrophages infiltrating the tumor microenvironment (TME), comprise 30–40% of the tumor mass in HGG and play a major role in promoting tumor angiogenesis and progression[5, 6]. Macrophage colony-stimulating factor 1 (CSF1) is a classical protumor cytokine that binds to the CSF1 receptor (CSF1R) to polarize macrophages in the TME from an antitumor M1-like phenotype toward the protumor M2 phenotype and promotes tumor angiogenesis, proliferation, metastasis, and immune escape[7, 8]. Inhibitors of CSF1R can inhibit the infiltration of macrophages in the TME, reshape their polarity, promote CD8^+^ T-cell infiltration, and prevent tumor progression[9]. A previous study showed that the TAM infiltration level was inversely correlated with survival in HGG patients treated with anti-VEGF therapy[10, 11]. Furthermore, M2 macrophage polarization has proven to be related to resistance to antiangiogenic treatments[12, 13]. These findings suggest that repolarizing the M1-like phenotype of macrophages is a potential therapeutic mechanism to reverse resistance to anti-VEGF therapy.

SYHA1813 is a selective TKI that inhibits VEGFR-1 (half maximal inhibitory concentration [IC_50_] = 2.8 nmol/L), VEGFR-2 (IC_50_ = 0.3 nmol/L), VEGFR-3 (IC_50_ = 4.3 nmol/L), and CSF1R (IC_50_ = 19.3 nmol/L) in kinase enzyme assays[14, 15]. The antitumor activity of SYHA1813 has been demonstrated in diverse xenograft models, and this activity was consistent with potent inhibition of angiogenesis and CSF1R signaling in a preclinical study (Shanghai Institute of Materia Medica, unpublished data, 2022).

The present study is a dose-escalation phase I clinical trial to evaluate the safety, pharmacokinetics (PK), maximum tolerated dose (MTD), efficacy, and potential biomarkers of SYHA1813 in patients with recurrent HGG or advanced solid tumors.

## Methods

### Patient eligibility

Eligible patients were ≥ 18 years of age with recurrent or advanced solid tumors confirmed by histology or cytology refractory to standard therapy or for which no effective therapy was available. Patients had at least one measurable lesion during the baseline period (primary central nervous system tumors were assessed as per the Response Assessment in Neuro-Oncology [RANO] criteria; other solid tumors were assessed as per Response Evaluation Criteria in Solid Tumors [RECIST] version 1.1). The time interval between the end of the last antitumor treatment and the first administration of SYHA1813 was ≥ four weeks for cytotoxic drugs, immunotherapy, macromolecular targeted drugs, and biological therapy; ≥ two weeks or five half-lives (whichever is longer) for oral small-molecule targeted drug therapy, anticancer traditional Chinese medicines or proprietary Chinese medicines; and ≥ four weeks for radiotherapy (≥ two weeks for palliative local radiotherapy for symptom relief). Other inclusion criteria included a Karnofsky Performance Status (KPS) of 60 or more for patients with central nervous system tumors, an Eastern Cooperative Oncology Group (ECOG) physical performance status of 0 to 2 for patients with other solid tumors, and a life expectancy of 12 weeks.

Exclusion criteria included participation in other interventional clinical studies within four weeks, major surgery within four weeks, use of glucocorticoids at a dose equivalent to more than 5 mg dexamethasone within five days for glioma or brain metastases, and use of bevacizumab, ramucirumab, and other anti-VEGF/VEGFR antibodies within three months for glioma or brain metastases. Patients were also excluded if they had impaired cardiac function or clinically significant cardiovascular and cerebrovascular diseases, including but not limited to a history of myocardial infarction, congestive heart failure, and unstable angina pectoris within six months; cerebrovascular accident within six months (patients with transient ischemic attack or lacunar infarction with no clinical significance could be enrolled); hypertension uncontrollable after medication (repeated blood pressure measurement at least 1 h apart, and blood pressure 150/90 mmHg at two consecutive tests); uncontrolled arrhythmia requiring medical treatment; QTc interval > 470 ms on electrocardiogram (ECG) examination; or left ventricular ejection fraction < 50%.

### Study design and drug administration

This was a phase Ia, multicenter, open-label, dose-escalation study of SYHA1813. The overall study design is presented in Supplementary Fig. S1. The primary endpoints were the safety and tolerability of SYHA1813, including the occurrence of dose-limiting toxicities (DLTs) and the establishment of the maximum tolerated dose (MTD). Secondary endpoints included determination of the PK profile and preliminary antitumor activity based on the objective response rate (ORR), and disease control rate (DCR), as well as exploration of potential biomarkers related to SYHA1813 therapy. The study was approved by the National Medical Products Administration (NMPA) for clinical trials. We conducted the study according to the ethical principles of the Declaration of Helsinki and Good Clinical Practices guidelines. The study was approved by an institutional review board at each participating site. All patients provided written informed consent. The study was registered at chictr.org.cn (trial registration ID: ChiCTR2100045380).

The maximum recommended starting dose (based body weight 60 kg) of 11 mg was estimated based on 1/6 of the highest non-severely toxic dose in the beagle dog study. Based on further safety considerations, 5 mg was selected as the starting dose in human subjects. In the dose-escalation study, patients were enrolled sequentially to receive a single oral dose of SYHA1813 (5, 15, 30, 60, 100, 150, and 200 mg) followed by a 3-day observation period with safety and PK assessments, subsequent once-daily treatment at the same dose level during 21-day multiple-dose period followed by a 4-day observation period with safety, PK and efficacy assessments. From the second cycle onwards, the study drug was given every day (3 weeks/cycle) if the treatment was well tolerated and beneficial.

### Safety assessments and definition of DLT

DLT was defined as the occurrence of adverse events (AEs) that the investigator judged to be related to SYHA1813 within 28 days of the first dose during the dose-escalation period (Supplementary Table S1). The MTD was defined as the maximum dose for which the probability of a DLT was ≤ 33%.

An accelerated titration design with one patient enrolled was utilized for the first two dose levels (5 and 15 mg). Then, a 3 + 3 dose-escalation design was employed starting from the third dose level (30 mg). If grade 3 or above adverse events (AEs, non-DLTs) occurred in one patient at the first two doses, two more patients were enrolled for further observation. If DLT was observed in one of the 3 patients, up to 3 more patients were enrolled at the same level. If 2 or more out of 3 to 6 patients experienced DLT at a dose level, the dose was decreased to the previous dose group. When decreasing to the previous dose group, if there were only 3 patients in the dose group, 3 more patients were added; if there were already 6 patients, the dose escalation ended, and the dose was defined as the MTD. Any patient who withdrew from the study before the completion of the DLT observation period due to a reason other than DLT was replaced. Dose de-escalation or reduction was permitted if safety re-evaluation was deemed necessary.

Safety analyses were conducted for all patients who received at least one dose of SYHA1813. AEs were assessed throughout the study using National Cancer Institute (NCI) Common Terminology Criteria for Adverse Events (CTCAE) version 5.0. The health status assessment of the patients included a physical examination (vital signs, weight, PS score, etc.), hematologic and biochemical profiling, routine urine and stool tests, and electrocardiogram assessment performed at screening and throughout the study.

### PK evaluation

Blood samples for the assessment of PK parameters were collected at predefined time points during the DLT observation period as follows: Cycle 0 Day 1 (predose; 0.5, 1, 2, 4, 8, and 12 h postdose), Day 2 (24 h postdose), and Day 3 (48 h postdose); Cycle 1 Day 1 (predose), Day 8 (predose), Day 15 (predose), Day 21 (predose; 0.5, 1, 2, 4, 8, and 12 h postdose), Day 22 (24 h postdose), Day 23 (48 h postdose), and Day 24 (72 h postdose). Plasma SYHA1813 levels were measured using a validated high-performance liquid chromatography‒mass spectrometry method.

Single- and multiple-dose PK parameters of SYHA1813 were estimated using noncompartmental analysis, including maximum observed plasma concentration (C_max_), C_max_/dose, time to C_max_ (T_max_), area under curve extrapolated to infinity (AUC_inf_), AUC_inf_/dose, volume of distribution (V_z_/F), terminal elimination half-life (t_1/2_), and oral clearance (CL/F) after the first dose. PK parameters at steady-state (ss), including C_max, ss_, C_max, ss_/dose, minimum observed plasma concentration (C_min, ss_), AUC_inf_, AUC_inf_/dose, T_max, ss_, V_ss_, t_1/2_, CL_ss_/F after multiple doses, and accumulation index (R_ac_), were also estimated.

### Efficacy assessment

Radiographic assessments were conducted at screening and at the end of the DLT observation period and repeated every six weeks during the extended treatment period until disease progression, intolerable toxicity, or withdrawal of consent (whichever came first). The patients with central nervous system tumors were evaluated for responses according to the RANO criteria by enhanced magnetic resonance imaging (MRI) scan. Patients with other solid tumors were assessed by computed tomography (CT) or MRI scan according to RECIST version 1.1. The categories used for the evaluation of the response to treatment included complete response (CR), partial response (PR), stable disease (SD), and progressive disease (PD). ORR and DCR were defined as CR + PR and CR + PR + SD, respectively.

### Exploratory biomarker analysis

Samples for biomarker analyses were collected on Cycle 1 Day 1 (predose), Cycle 1 Day 24 (72 h post-dose), and 14 days after the end of treatment. Soluble VEGFR2, VEGF, and CSF1 levels were measured using multiplex enzyme-link immunosorbent assay (ELISA) plates from R&D Systems (Minneapolis, MN), and placental growth factor (PlGF) levels were measured using an electrochemiluminescence immunoassay (ECLIA) kit from Roche Diagnostics (Mannheim, Germany).

### Statistical analysis

All statistical analyses were performed using SAS® 9.4 (SAS Institute, Inc., Cary, NC, USA) except for the calculation of PK parameters using Phoenix® WinNonlin 8.1 (Pharsight Corp., Certara, Princeton, NJ, USA). Exploratory biomarker analyses were performed by a two-tailed paired t test using GraphPad Prism 9.3 (GraphPad Software, Inc., CA, USA).

## Results

### Patients and treatments

This dose-escalation study was conducted at 4 study centers in China starting on May 26, 2021. As of the data cutoff date of February 15, 2022, 14 patients had received SYHA1813, including 1 patient in the 5-mg group, 8 patients in the 15-mg group, and 5 patients in the 30-mg group. Ten patients remained in the study for the entire DLT observation period (Supplementary Fig. S2). Reasons for discontinuing treatment were AEs (n = 3), withdrawal of consent (n = 2), DLT (n = 2), and investigator discretion (n = 1). The baseline characteristics of the patients are summarized in Table 1. The median age was 46 years, and 64.3% of the patients were male. Among the 14 enrolled patients, 4 patients were diagnosed with WHO grade IV glioblastoma, 4 with WHO grade III astrocytoma, 2 with WHO grade III oligodendroglioma, 2 with WHO grade II-III astrocytoma, 1 with WHO grade III to IV glioma, and 1 with colorectal cancer.

**Table 1:** Patient demographics and baseline characteristics

Characteristics	5 mg (n = 1)	15 mg (n = 8)	30 mg (n = 5)	All (n = 14)
Age, years				
Median	33.0	47.4	46.4	46.0
Range	33–33	39–52	34–69	33–69
Sex, n (%)				
Male	1 (100.0)	4 (50.0)	4 (80.0)	9 (64.3)
Female	0	4 (50.0)	1 (20.0)	5 (35.7)
Tumor type, n (%)				
High-grade glioma	1 (100.0)	7 (87.5)	5 (100.0)	13 (92.9)
WHO grade III astrocytoma	1 (100.0)	2 (25.0)	1 (20.0)	4 (28.6)
WHO grade IV glioblastoma	0	1 (12.5)	3 (60.0)	4 (28.6)
WHO grade III oligodendroglioma	0	2 (25.0)	0	2 (14.3)
WHO grade II to III astrocytoma	0	2 (25.0)	0	2 (14.3)
WHO grade III to IV glioma	0	0	1 (20.0)	1 (7.1)
Solid tumor	0	1 (12.5)	0	1 (7.1)
Colorectal cancer	0	1 (12.5)	0	1 (7.1)
Prior systemic therapy, n (%)				
1	0	^a^2 (25.0)	1 (20.0)	3 (21.4)
2	1 (100.0)	1 (12.5)	2 (40.0)	4 (28.6)
≥ 3	0	5 (62.6)	2 (40.0)	7 (50.0)

^a^Note: one patient did not have data regarding history of prior antitumor treatments.

### DLTs and MTD

According to the accelerated titration design, two patients successively completed the DLT observation period (28 days) of treatment at 5 and 15 mg. However, the patient treated with 15 mg experienced grade 3 hypertension (non-DLT), and another two patients were enrolled at 15 mg based on the study design. Of those patients, one patient discontinued the treatment due to grade 2 bradycardia, which the investigator considered not related to the treatment. As a result, one more patient was enrolled at 15 mg for safety evaluation, and no more than grade 3 AEs were reported. In the 30 mg group, two out of five patients experienced DLTs (grade 3 mucositis oral and grade 4 hypertension), and the drug administration to the sixth patient in the 30 mg group was stopped according to the investigator’s decision to protect the safety and welfare of patients. Hence, for safety re-evaluation, four additional patients were enrolled in the lower dose level of 15 mg (one patient withdrew consent and was replaced), and no DLTs occurred. The dose-escalation process is shown in Supplementary Fig. S3.

Two patients in the dose escalation had DLTs. The first patient with WHO grade III anaplastic astrocytoma in the 30-mg group had a DLT (grade 3 mucositis oral) on Cycle 1 Day 18, which lasted 5 days. The second patient with WHO grade IV glioblastoma in the 30-mg group had a DLT (grade 4 hypertension) on Cycle 1 Day 15, which lasted 8 days. No DLT was observed at doses of 15 mg and lower. Based on protocol-defined criteria, the MTD for SYHA1813 was determined to be 15 mg once daily.

### Safety and tolerability

All 14 patients were evaluated for safety. Overall, 13 (92.9%) patients had treatment-emergent AEs (TEAEs) during the study (Table 2). The most common TEAEs were hypertension (42.9%), sinus bradycardia (42.9%), platelet count decreased (35.7%), blood triglyceride increased (35.7%), alanine aminotransferase increased (28.6%), aspartate aminotransferase increased (28.6%), and urinary tract infection (28.6%). The majority of TEAEs were ≤ grade 2 and resolved spontaneously after drug discontinuation. Grade ≥ 3 TEAEs, including hypertension (35.7%), platelet count decreased (14.3%), and mucositis oral (7.1%), were all resolved after treatment withdrawal. There were 11 (78.6%) patients who experienced treatment-related AEs (TRAEs) (Supplementary Table S2).

**Table 2:** Treatment-emergent adverse events (affecting ≥ 10% of patients in either treatment group)

MedDRA-Preferred Term, n (%)	5 mg (n = 1)		15 mg (n = 8)		30 mg (n = 5)		All (n = 14)	
	Any grade	Grades 3–4	Any grade	Grades 3–4	Any grade	Grades 3–4	Any grade	Grades 3–4
Laboratory abnormalities								
Platelet count decreased	0	0	1 (12.5)	0	4 (80.0)	2 (40.0)	5 (35.7)	2 (14.2)
Blood triglyceride increased	0	0	3 (37.5)	0	2 (40.0)	0	5 (35.7)	0
ALT increased	0	0	2 (25.0)	0	2 (40.0)	0	4 (28.6)	0
AST increased	0	0	2 (25.0)	0	2 (40.0)	0	4 (28.6)	0
Blood LDH increased	0	0	2 (25.0)	0	1 (20.0)	0	3 (21.4)	0
α-HBDH increased	0	0	2 (25.0)	0	0	0	2 (14.3)	0
Hyperlipidemia	0	0	1 (12.5)	0	2 (40.0)	0	3 (21.4)	0
Hypokalemia	0	0	3 (37.5)	0	0	0	3 (21.4)	0
Cholesterol high	0	0	2 (25.0)	0	0	0	2 (14.3)	0
Neutrophil count decreased	0	0	1 (12.5)	0	1 (20.0)	0	2 (14.3)	0
Lymphocyte count decreased	0	0	1 (12.5)	0	1 (20.0)	0	2 (14.3)	0
White blood cell decreased	0	0	1 (12.5)	0	1 (20.0)	0	2 (14.3)	0
Proteinuria	0	0	0	0	2 (40.0)	0	2 (14.3)	0
Clinical adverse events								
Hypertension	0	0	2 (25.0)	1 (12.5)	4 (80.0)	4 (80.0)	6 (42.9)	5 (35.7)
Sinus bradycardia	0	0	2 (25.0)	0	4 (80.0)	0	6 (42.9)	0
Urinary tract infection	1 (100.0)	0	1 (12.5)	0	2 (40.0)	0	4 (28.6)	0
Mucositis oral	0	0	0	0	2 (40.0)	1 (20.0)	2 (14.3)	1 (7.1)
Diarrhea	0	0	0	0	2 (40.0)	0	2 (14.3)	0
Epilepsy	0	0	0	0	2 (40.0)	0	2 (14.3)	0
Hydrocephalus	0	0	1 (12.5)	0	1 (20.0)	0	2 (14.3)	0
Headache	0	0	0	0	2 (40.0)	0	2 (14.3)	0

Abbreviations: ALT, alanine aminotransferase; AST, aspartate aminotransferase; LDH, lactate dehydrogenase; α-HBDH, alpha-hydroxybutyrate-dehydrogenase.

Four patients experienced treatment interruptions and dose reductions due to SYHA1813-related toxicities, which were grade 2 sinus bradycardia, grade 2 platelet count decreased, grade 3 mucositis oral, and grade 4 hypertension. Serious adverse events considered to be related to SYHA1813 treatment were reported for one (7.1%) patient with grade 4 hypertension in the 30-mg group. No grade 5 AEs occurred.

### PK

**Fig. 1:**
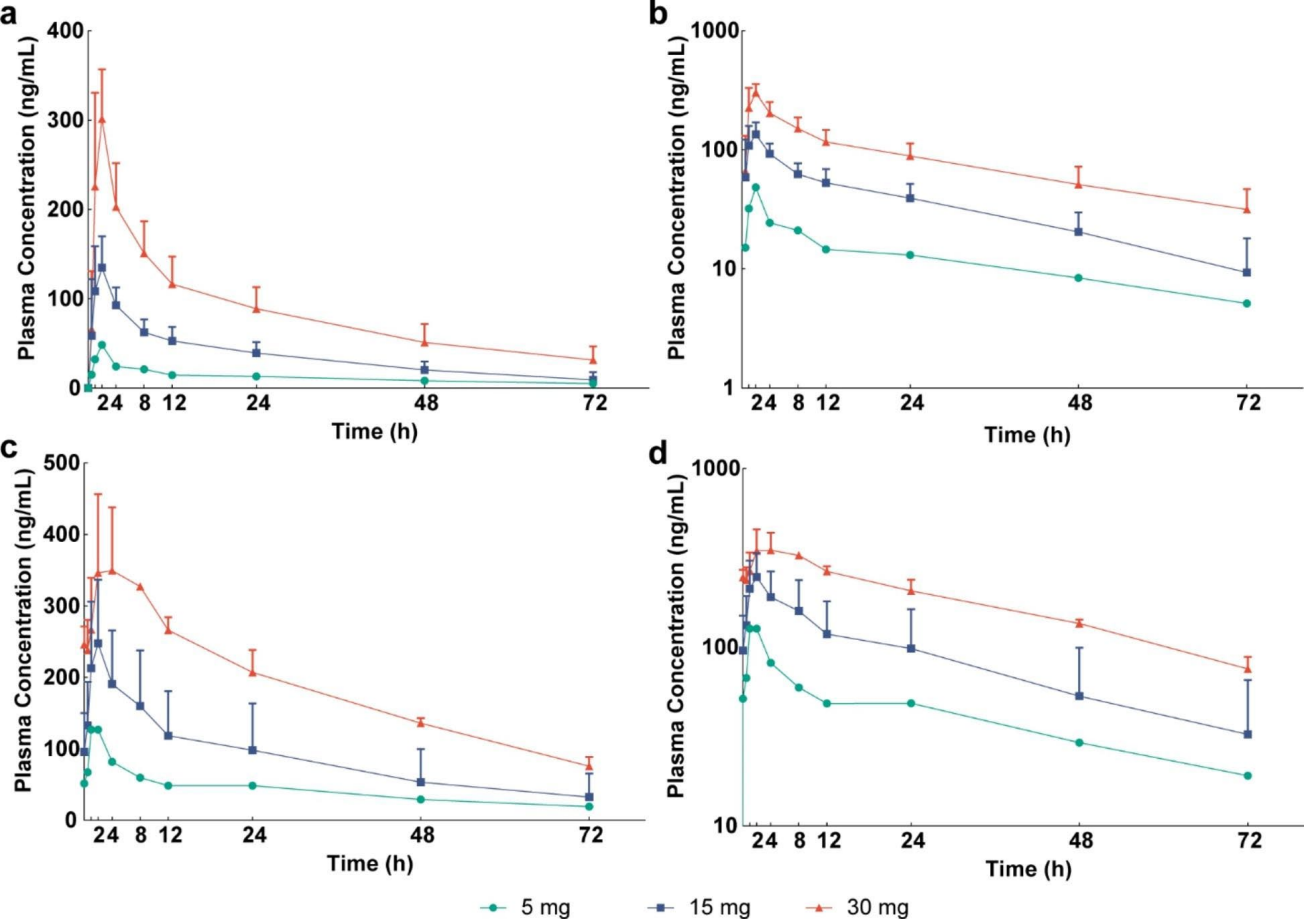
Pharmacokinetic profiles. Mean (SD) plasma concentrations of SYHA1813 versus time: **a** single dose (linear scale); **b** single dose (semilog scale); **c** multiple doses (linear scale); **d** multiple doses (semilog scale)

The plasma PK parameters are summarized in Supplementary Table 3. The mean plasma concentration-time curves of SYHA1813 were plotted after a single dose (Fig. 1a, b) and multiple doses (Fig. 1c, d) at 5, 15, and 30 mg. After single-dose administration ranging from 5 mg to 30 mg, SYHA1813 exhibited rapid absorption with median T_max_ values of 2.0 h, independent of dose. Subsequently, the concentration declined slowly; the mean t_1/2_ was approximately 26.5 to 36.8 h. The mean apparent volume of distribution (V_z_/F) and apparent clearance (CL/F) were 188.8 ~ 231.0 L and 4.3 ~ 5.6 L/h, respectively. The C_max_ and AUC_inf_ increased with increasing doses and generally demonstrated linear PK properties. At steady state, the C_max, ss_ was reached after approximately 1.0 ~ 8.0 h, and the mean t_1/2_ was 27.6 ~ 35.7 h. In addition, the accumulation ratio R_ac_ (C_max_) and R_ac_ (AUC) of SYHA1813 ranged from 1.1 to 2.6 and 1.8 to 3.3, respectively, suggesting weak to moderate accumulation[16].

**Table 3:** Pharmacokinetic Parameters After a Single Oral Dose and Multiple Oral Doses of SYHA1813

Parameter	Dose					
	5 mg		15 mg		30 mg	
	Mean	SD	Mean	SD	Mean	SD
A single dose						
No. of patients	1		8		5	
C_max_, ng/mL	48.5	/	148.9	32.1	301.4	55.6
C_max_/dose, ng/mL/mg	9.7	/	9.9	2.1	10.0	1.9
^a^T_max_, h	2.0	2.0	2.0	0.5–2.1	2.0	2.0-2.1
t_1/2_, h	36.8	/	26.5	6.6	30.9	6.2
AUC_inf_, h·ng/mL	1149.9	/	3040.3	1172.0	7421.7	2788.1
AUC_inf_/dose, h·ng/mL/mg	230.0	/	202.7	78.1	247.4	92.9
V_z_/F, L	231.0	/	199.7	45.2	188.8	35.4
CL/F, L/h	4.3	/	5.6	2.1	4.4	1.2
At steady state						
No. of patients	1		5		3	
C_min, ss_, ng/mL	48.5	/	94.3	55.1	206.0	31.0
C_max, ss_, ng/mL	127.0	/	248.0	88.9	376.0	80.6
C_max, ss_/dose, ng/mL/mg	25.4	/	16.5	5.9	12.5	2.7
^a^T_max, ss_, h	1.0	1.0	2.0	0.9-2.0	8.0	2.0–8.0
t_1/2_, h	35.7	/	27.6	6.9	32.9	1.5
AUC_ss_, h·ng/mL	1487.5	/	3354.1	1651.2	6599.5	783.5
AUC_inf_/dose, h·ng/mL/mg	791.5	/	511.9	395.2	557.2	55.6
V_ss_, L	173.1	/	196.9	59.2	218.0	26.2
CL_ss_/F, L/h	3.4	/	5.3	2.3	4.6	0.5
R_ac_ (C_max_)	2.6	/	1.7	0.3	1.1	0.0
R_ac_ (AUC)	3.3	/	2.3	0.5	1.8	0.3

Abbreviations: SD, standard deviation; C_max_, maximum plasma drug concentration after single-dose administration; C_min_, _ss_, minimum steady-state drug concentration in plasma; T_max_, time to maximum concentration; t_1/2_, terminal elimination half-life; AUC_inf_, area under plasma concentration-time curve from zero to infinity; AUC_ss_, area under plasma concentration-time curve at steady-state; V_z_/F, volume of distribution; V_ss_, volume of distribution at steady-state; CL/F, apparent oral clearance; CL_ss_/F, apparent oral clearance at steady-state; R_ac_, accumulation ratio index.

^a^T_max_ (h) is presented as the median and range.

### Efficacy

Ten patients were assessable for tumor response per investigator assessment, 9 with glioma (RANO criteria) and 1 with colorectal cancer (RECIST version 1.1); the other four enrolled patients withdrew from the study before the first tumor assessment. In the efficacy-evaluable population, the ORR was 20% (2/10), with all responses being PRs (Supplementary Table S3).

**Fig. 2:**
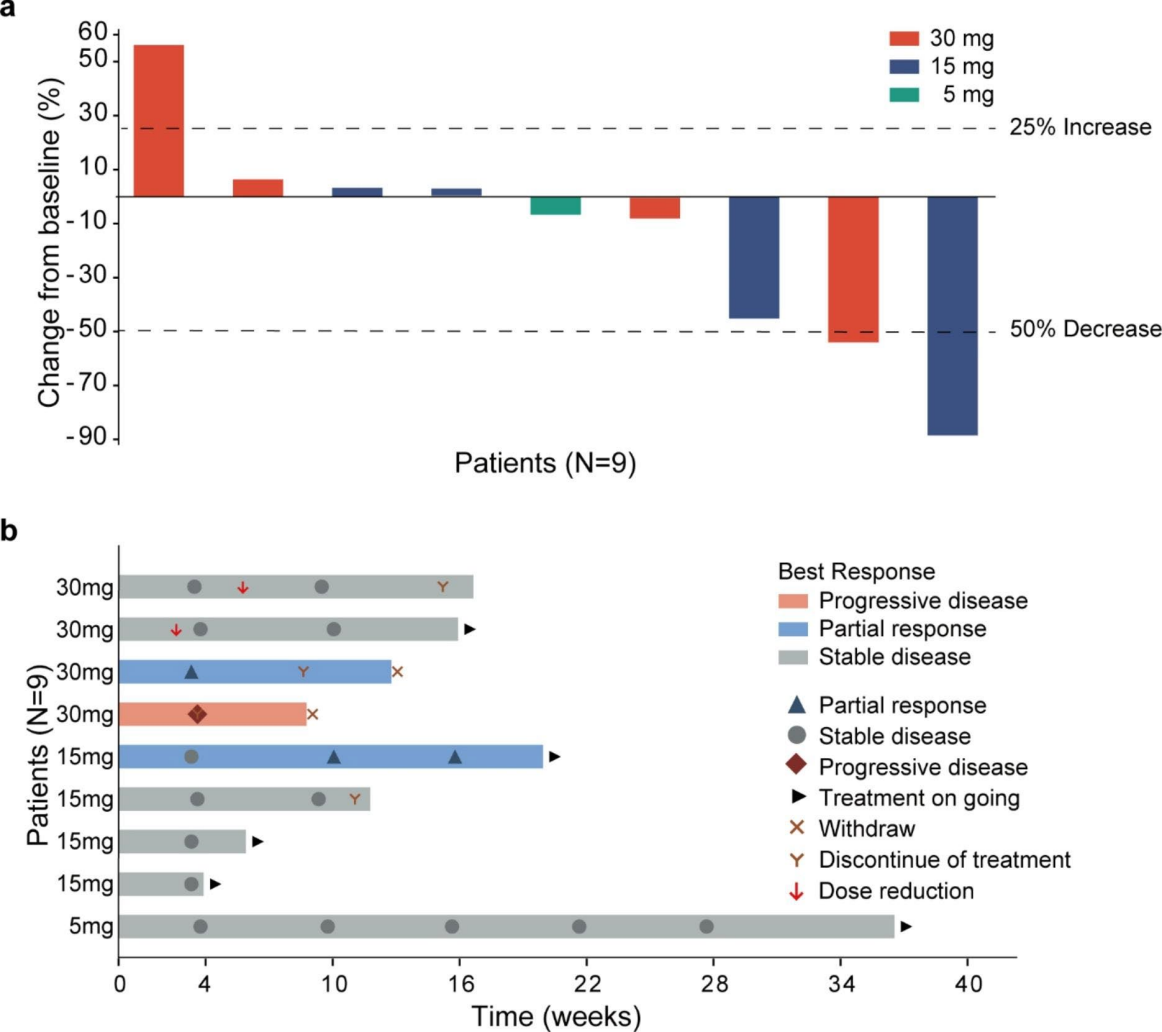
Antitumor activity for patients with HGG. **a** Best response in target lesion. The dotted lines at − 50% and 25% indicate boundaries for response and progression, respectively. **b** Treatment duration, time to best overall response, and time to first progression. Each bar represents an individual patient

For glioma, the best percent change in tumor size from baseline and the best response and time on therapy for patients with HGG are shown in Fig. 2a and b. Two patients achieved PR: one with WHO grade III astrocytoma in the 15-mg group and one with WHO grade IV glioblastoma in the 30-mg group. Seven (70%) patients achieved SD. One patient with anaplastic astrocytoma (WHO grade III) achieved prolonged SD longer than six months in the 5-mg group. One (10%) patient with WHO grade III astrocytoma developed PD. The overall response and disease control rates were 20% and 90%, respectively. An example of objective response in one patient with WHO grade III astrocytoma treated at 15 mg is shown in Supplementary Fig. S4. The tumor volume score decreased by 88.4% in this patient after four months of treatment, indicating a substantial tumor burden reduction. Alleviation of cerebral edema persisted for at least three months, as shown in axial FLAIR MRI images (Supplementary Fig. S5).

The other advanced solid tumor patient, a 49-year-old male patient with stage IV colorectal cancer, received standard regimens and developed PD. He was then enrolled in the 15-mg SYHA1813 group and achieved SD assessed on Cycle 1 Day 25, which had lasted 2 weeks by the study cutoff date.

### Analysis of biomarkers

Five patients were excluded from the biomarker analysis set due to a lack of postdose measurements. After multiple-dose administration of SYHA1813, there were significant increases in the serum levels of PlGF (*P* = .0484, Supplementary Fig. S6a) and VEGFA (*P* = .0092, Supplementary Fig. S6b). Compared with those at baseline, the levels of sVEGFR2 were significantly decreased by Day 24 (*P* = .0023, Supplementary Fig. S6c). The level of serum CSF1 increased in 7 of 9 (77.8%) patients (Supplementary Fig. S6d).

## Discussion

This trial was the first to investigate the safety, PK profile, and preliminary antitumor activity of single and multiple doses of SYHA1813. The MTD of SYHA1813 was defined as 15 mg once daily.

While most patients experienced at least one TRAE, most treatment-related toxicities were mild and included hypertension, platelet count decreased, and sinus bradycardia. Doses up to 15 mg per day were considered tolerable. Two patients in the 30-mg group experienced DLTs. Hypertension and platelet count decreased were the most frequently reported grade 3–4 AEs. The occurrence of hypertension was likely the result of effective inhibition of the VEGF signaling pathway, consistent with the observations of increased incidence of hypertension in other clinical studies with small-molecule VEGF TKIs[17]. This effect is considered to be mediated by the disruption of nitric oxide-activated VEGFR2 signaling, which can result in physiological vasodilation[18]. Close monitoring and timely adjustment of blood pressure according to standard blood pressure treatment guidelines are recommended in future studies. Platelets as carriers of VEGF have been suggested to play a role as regulators of endothelial cell function, possibly affected during treatment with SYHA1813 [19, 20]. In general, the on-target toxicities caused by SYHA1813 were hypertension, mucositis oral, and platelet count decreased, similar to those caused by other small molecular VEGFR TKIs [20–23].

When administered as a single dose, the exposure (C_max_ and AUC_inf_) of SYHA1813 increased approximately linearly over the studied dose range. The results of the current study established the MTD of SYHA1813 as 15 mg once daily. After multiple doses of 5 mg SYHA1813, the unbound trough concentration at steady state was much higher than the in vitro IC_50_ of VEGFRs and CSF1R, supporting that a daily oral dose of 5 mg and above is expected to be efficacious. Thus, 15 mg was selected as the recommended dose for the dose-expansion cohort to further determine the therapeutic activity of SYHA1813.

SYHA1813 administration resulted in a significant decrease in the level of sVEGFR2 from baseline, consistent with previous clinical findings with other VEGFR inhibitors, such as cediranib and pazopanib. Previous preclinical studies suggested that changes in the level of sVEGFR2 could be a pharmacodynamic marker of systemic exposure to drug, not a predictive marker of tumor response or clinical benefit. Similar to a previous study of the CSF1R inhibitor pexidartinib, we also observed an increased plasma concentration of CSF1 following multiple-dose administration [24]. A major implication of such an increase in CSF1 level is that there was strong on-target inhibition of CSF1R [25].

SYHA1813 has promising antitumor activity in recurrent HGG. A sustained tumor response was observed in HGG. Similar to the results of the phase II studies of cediranib (ORR of 56.7%) and pazopanib (ORR of 5.9%) in patients with recurrent malignant gliomas, SYHA1813 monotherapy showed encouraging antitumor activity (ORR of 20%) [4, 22]. Our findings are in line with the therapeutic mechanisms SYHA1813 was designed to have. Neoangiogenesis is a critical feature of glioblastoma, and several VEGFR-targeting small molecules have been extensively investigated for the treatment of glioblastoma [26]. However, exploration of multitarget TKIs such as cediranib and pazopanib as monotherapy have failed to improve the survival of recurrent glioblastoma patients [4, 21, 22]. CSF1R is an interesting target because it is overexpressed in HGG; and its levels are associated with glioma grade, and high CSF1R levels are associated with poor clinical outcomes [27–29]. Although pexidartinib (a TKI of CSR1R) is approved by the FDA based on its significant improvement of ORR, it did not show meaningful improvement in progression-free survival compared with previous data in recurrent glioblastoma [9]. The inhibition of multiple VEGFRs and CSF1R by SYHA1813 may lead to synergistic effects through the immunoregulation of macrophages to potentially overcome resistance to anti-VEGFR treatment and enhance antitumor activity.

In conclusion, oral administration of SYHA1813 at 15 mg daily demonstrated acceptable tolerability and preliminary antitumor activity in recurrent HGG. The subsequent dose-expansion cohort and phase Ib clinical trials of SYHA1813 should include HGG as a focus.

## Electronic supplementary material

Below is the link to the electronic supplementary material.

Supplementary Material 1
