# Supplementary material for: A phase I dose-escalation study of SYHA1813, a VEGFR and CSF1R inhibitor, in patients with recurrent High-Grade Gliomas or Advanced Solid Tumors
Source: Invest New Drugs. 2023 Mar 8;41(2):296–305. doi: 10.1007/s10637-022-01325-4 (PMC10140125; doi:10.1007/s10637-022-01325-4)
Supplement: Supplementary file 1 — Supplementary Material 1 [file 10637_2022_1325_MOESM1_ESM.docx]

Supplementary Material

# Supplementary Figures


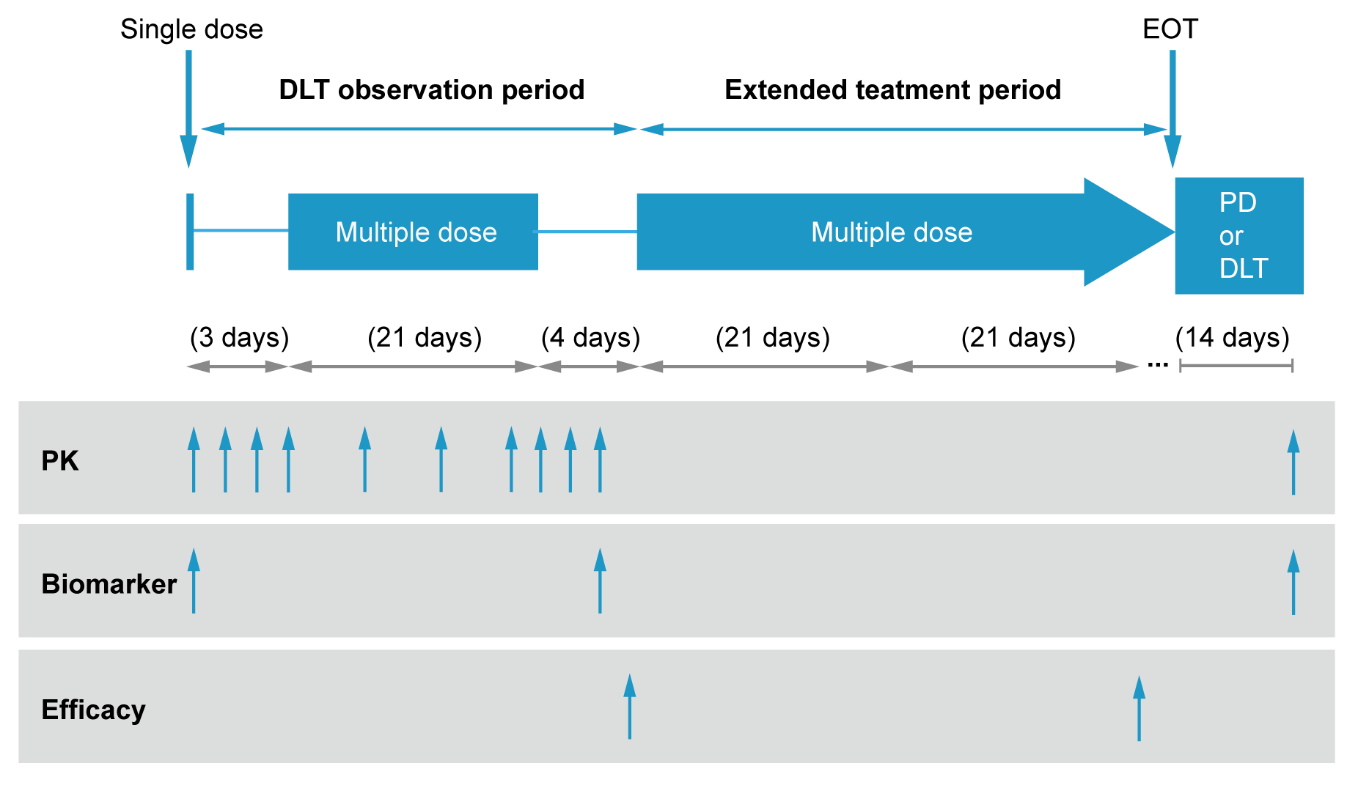


**Fig. S1** Study design. EOT, end of treatment; DLT, dose-limiting toxicity; PD, progressive disease; PK, pharmacokinetics. The dose-escalation study consisted of 4 periods: screening (up to 28 days), DLT observation (28 days), extended treatment (3 weeks/cycle), and follow-up. The DLT observation period included a 3-day single-dose period (Cycle 0), a 21-day multiple-dose period (Cycle 1 Day 1 to Day 21), and a 4-day safety observation period (Cycle 1 Day 22 to Day 25). The study drug was given on the first day of the single-dose period (Cycle 0 Day 1) and every day during the multiple-dose period. The first cycle of the extended treatment period was denoted as Cycle 2.


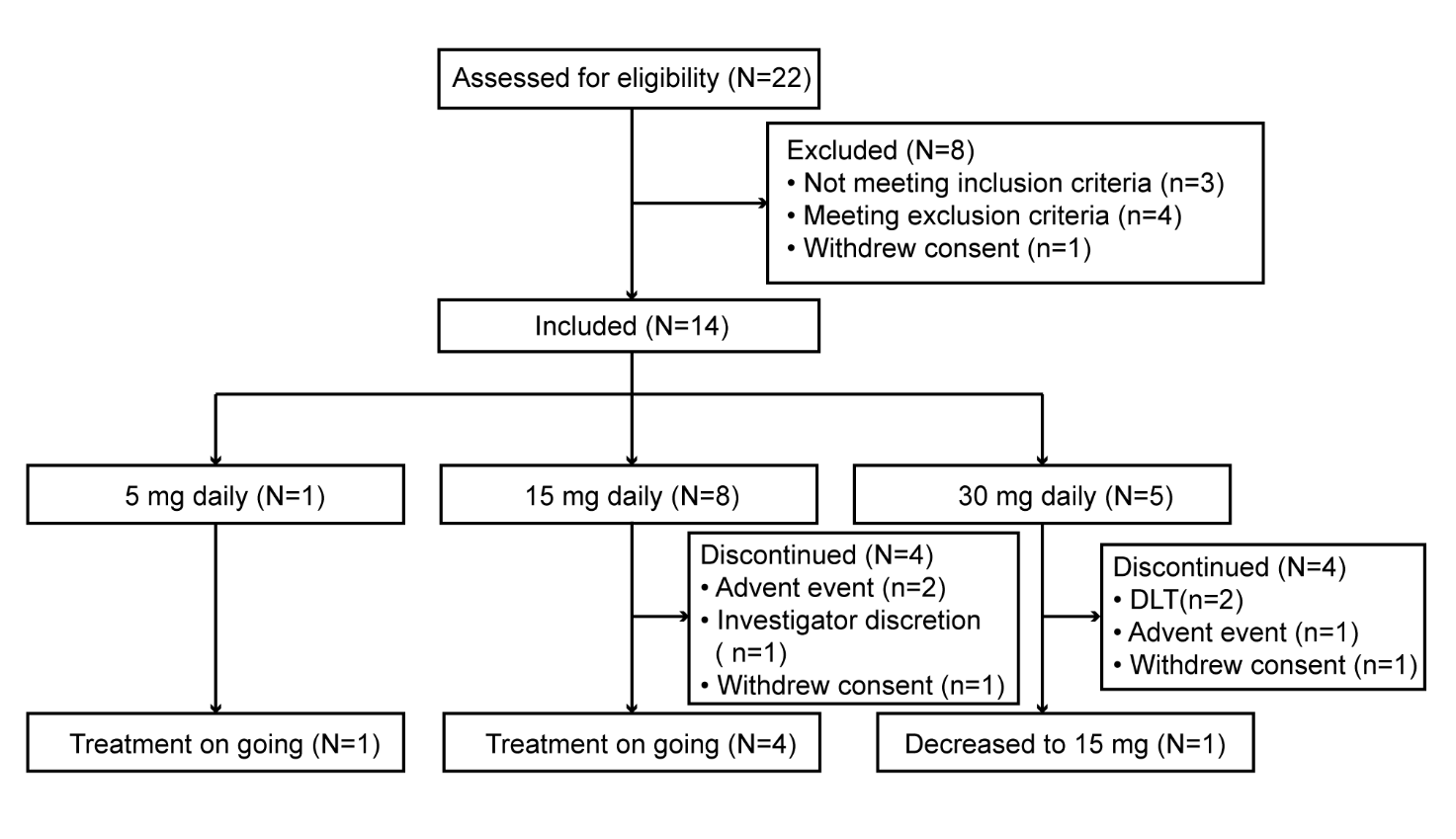


**Fig. S2** Patient disposition


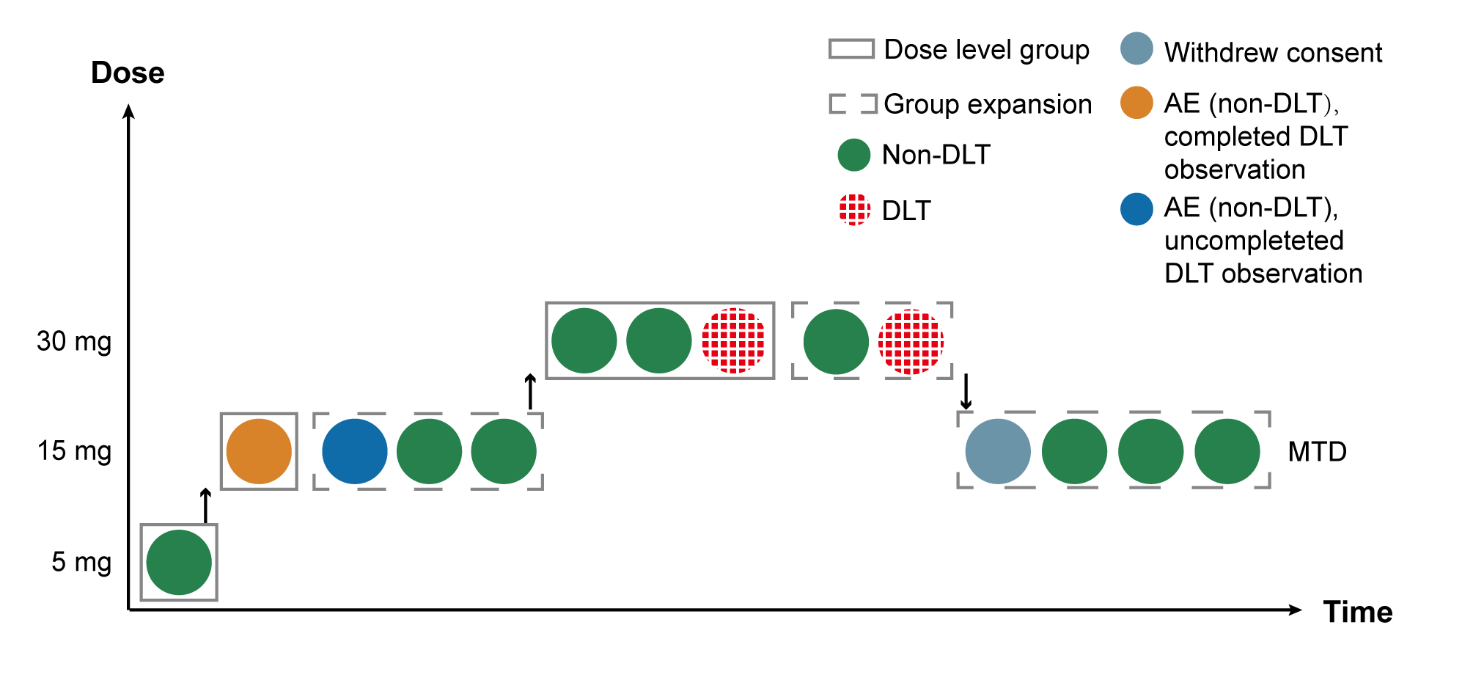


**Fig. S3** Dose-escalation process.


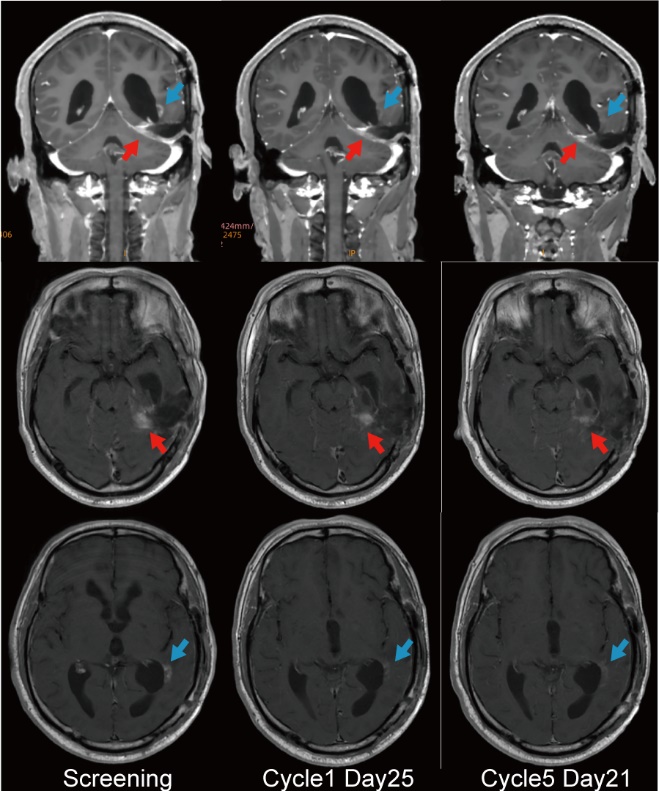


**Fig. S4** Representative MRI images of a patient with Objective Response. The baseline MRI shows a target lesion (red arrow) that measured perpendicular diameters of 17.5 mm and 11.2 mm in the left temporal lobe. The tumor decreased in volume by 36.2% at Cycle1 Day25 and by 88.4% at Cycle 5 Day 21, which reflects the RANO criteria of stable disease and partial response. A non-target lesion (blue arrow) in the baseline image and follow-up images that complete disappearance at Cycle 5 Day 21.


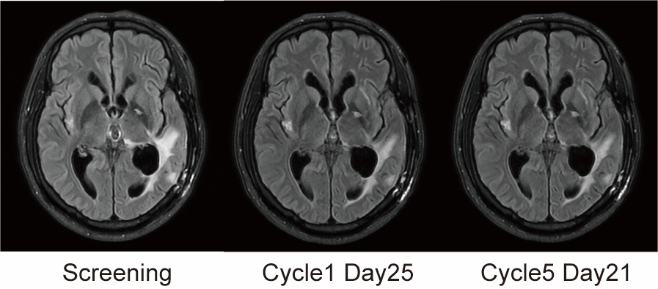


**Fig. S5** Representative FLAIR MRI images to show the reduction in cerebral edema by SYHA1813.


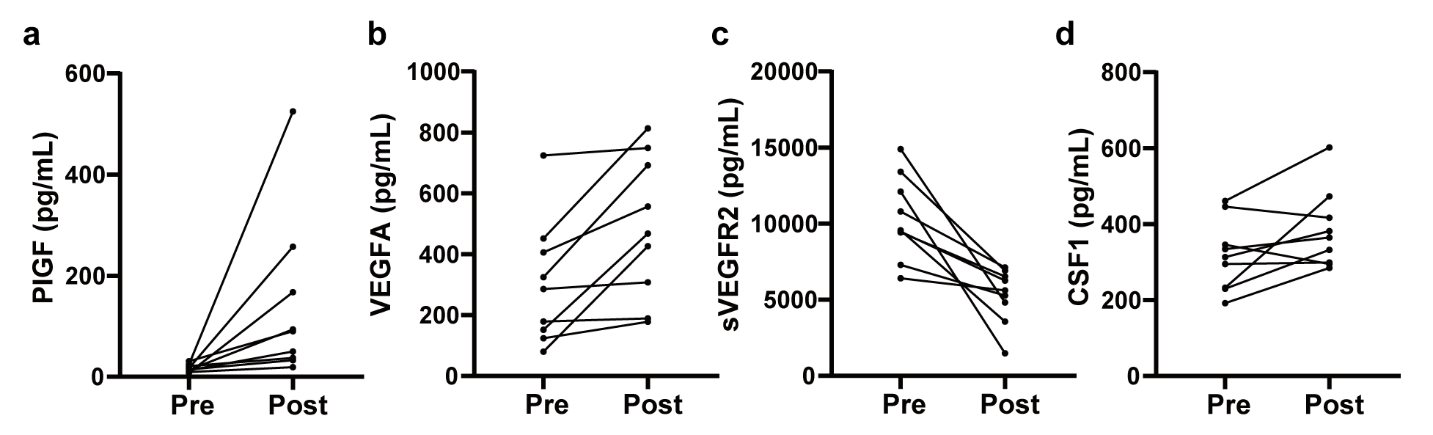


**Fig.S6** Post-treatment changes in plasma PlGF (**a**), VEGFA (**b**), sVEGFR2 (**c**), and CSF1 (**d**) for individual patients.

# Supplementary Tables

**Supplementary Table S1.** DLT definition

| **Toxicity** | **Occurrence of any of the following AE** |
| --- | --- |
| Hematological toxicity | - Febrile neutropenia (absolute neutrophil count < 1.0×10^9^ /L with a single temperature > 38.3°C or a sustained temperature ≥ 38°C for more than 1 h) ≥ 7 days; - Grade 4 neutropenia lasting ≥ 7 days (two above allow to use therapeutic colony-stimulating factors); - Grade 4 thrombocytopenia; - Grade 3 thrombocytopenia with bleeding. |
| Non-hematological toxicity | Any other grade ≥ 3 non-hematological AE except for the following:   - ≥ Grade 3 neurological events in patients with brain tumor, possibly caused by the brain lesion, brain surgery or anticipated brain postoperative complication; - Any grade Seizure in patients with brain tumor; - ≥ Grade 3 thrombotic events after brain tumor surgery; - Grade 3 transient abdominal pain (≤ 24 hours), headache and fever, etc., recovered to Grade 2 or baseline level after treatment; - Grade 3 electrolyte disturbance/appetite/nausea/vomiting/diarrhea/fatigue, relieved to ≤ Grade 2 or baseline level within 3 days after best support care; - Grade 3 abnormal liver function, recovered to grade 2 or baseline level within 7 days after treatment; - Grade 3 elevated blood pressure/proteinuria, relieved spontaneously or drug-controlled to ≤ grade 2 within 3 days; - Allergic reaction; - Other Grade 3 toxicities, recovered to ≤ grade 2 within 3 days without special treatment or after supportive treatment. |

**Supplementary Table S2.** Treatment-related adverse events (affecting ≥ 10% of patients in either treatment group)

| **MedDRA-Preferred Term, n (%)** | **5 mg (n=1)** | | **15 mg (n=8)** | | **30 mg (n=5)** | | **All (n=14)** | |
| --- | --- | --- | --- | --- | --- | --- | --- | --- |
|  | **Any grade** | **Grades 3-4** | **Any grade** | **Grades 3-4** | **Any grade** | **Grades 3-4** | **Any grade** | **Grades 3-4** |
| Laboratory abnormalities | | | | | | | | |
| Platelet count decreased | 0 | 0 | 1 (12.5) | 0 | 2 (40.0) | 2 (40.0) | 5 (35.7) | 2 (14.2) |
| ALT increased | 0 | 0 | 2 (25.0) | 0 | 2 (40.0) | 0 | 4 (28.6) | 0 |
| AST increased | 0 | 0 | 1 (12.5) | 0 | 2 (40.0) | 0 | 3 (21.4) | 0 |
| Blood LDH increased | 0 | 0 | 2 (25.0) | 0 | 1 (20.0) | 0 | 3 (21.4) | 0 |
| α-HBDH increased | 0 | 0 | 2 (25.0) | 0 | 0 | 0 | 2 (14.3) | 0 |
| Neutrophil count decreased | 0 | 0 | 1 (12.5) | 0 | 1 (20.0) | 0 | 2 (14.3) | 0 |
| Lymphocyte count decreased | 0 | 0 | 1 (12.5) | 0 | 1 (20.0) | 0 | 2 (14.3) | 0 |
| White blood cell decreased | 0 | 0 | 1 (12.5) | 0 | 1 (20.0) | 0 | 2 (14.3) | 0 |
| Hypertriglyceridemia | 0 | 0 | 2 (25.0) | 0 |  | 0 | 2 (14.3) | 0 |
| Cholesterol high | 0 | 0 | 2 (25.0) | 0 | 0 | 0 | 2 (14.3) | 0 |
| Hyperlipidemia | 0 | 0 | 1 (12.5) | 0 | 1 (20.0) | 0 | 2 (14.3) | 0 |
| Proteinuria | 0 | 0 | 0 | 0 | 2 (40.0) | 0 | 2 (14.3) | 0 |
| Clinical adverse events | | | | | | | | |
| Hypertension | 0 | 0 | 2 (25.0) | 1 (12.5) | 4 (80.0) | 4 (80.0) | 6 (42.9) | 5 (35.7) |
| Sinus bradycardia | 0 | 0 | 2 (25.0) | 0 | 3 (60.0) | 0 | 5 (35.7) | 0 |
| Mucositis oral | 0 | 0 | 0 | 0 | 1 (20.0) | 1 (20.0) | 2 (14.3) | 1 (7.1) |
| Abbreviations: ALT, alanine aminotransferase; AST, aspartate aminotransferase; LDH, lactate dehydrogenase; α-HBDH, alpha-hydroxybutyrate-dehydrogenase. | | | | | | | | |

**Supplementary Table S3.** Clinical response to SYHA1813 therapy

|  | **5 mg**  **(n=1)** | **15 mg**  **(n=5)** | **30 mg**  **(n=4)** | **All**  **(n=10)** |
| --- | --- | --- | --- | --- |
| Best overall response, n (%) |  |  |  |  |
| Complete response | 0 | 0 | 0 | 0 |
| Partial response | 0 | 1 (20) | 1 (25) | 2 (20) |
| Stable disease | 1 (100) | 4 (80) | 2 (50) | 7 (70) |
| Progressive disease | 0 | 0 | 1 (25) | 1 (10) |
| Overall response rate (CR/PR), n (%) | 0 | 1 (20) | 1 (25) | 2 (20) |
| Disease control rate (CR/PR/SD), n (%) | 1 (100) | 5 (100) | 3 (75) | 9 (90) |
| Abbreviations: CR, complete response; PR, partial response; SD, stable disease. Note. Central nervous system tumors were evaluated according to the RANO standard and solid tumors were measured according to RECIST version 1.1 criteria. | | | | |
